# Endoscopic-assisted strip craniectomy using a diamond drill bit attached to a dural retractor: a technical paper

**DOI:** 10.1007/s00381-026-07227-7

**Published:** 2026-04-09

**Authors:** Joyce Koueik, Catharine B. Garland, Anas Abou Merhi, Sarah Larson, Jordan Henry, Daniel Y. Cho, Raheel Ahmed, Bermans J. Iskandar

**Affiliations:** 1 Departments of Neurological SurgerySchool of Medicine and Public HealthClinical Science Center, University of Wisconsin, 600 Highland Avenue, Madison, WI 53792-8660 USA; 2 Department of Surgery, Division of Plastic and Reconstructive Surgery, School of Medicine and Public Health, University of Wisconsin, Madison, WI USA

**Keywords:** Craniosynostosis, Minimal invasive, Suturectomy, Blood loss

## Abstract

**Background and importance:**

Minimally invasive techniques have largely replaced open cranial vault remodeling as the preferred treatment approach for infants with single-suture craniosynostosis. We describe a refined method for minimizing blood loss during strip craniectomy, using a high-speed drill with a diamond burr and a novel attachable dural retractor. The diamond drill bit generates localized heat that cauterizes the bone edges, thereby potentially reducing bleeding, while the dural retractor is designed to limit heat dissipation and protects the dura from thermal and mechanical injury.

**Clinical presentation:**

A 3-month-old female infant with trigonocephaly secondary to metopic craniosynostosis underwent endoscopy-assisted metopic strip craniectomy, followed by postoperative cranial molding helmet therapy. The accompanying video demonstrates the operative technique and key steps of the procedure.

**Conclusion:**

This technical note demonstrates the feasibility of reducing bone-edge blood loss during endoscopic strip craniectomy using a diamond burr equipped with an attachable dural retractor. Further evaluation in larger cohort studies is necessary to objectively assess safety, reproducibility, and impact on perioperative blood loss.

**Supplementary Information:**

The online version contains supplementary material available at 10.1007/s00381-026-07227-7.

## Background and importance

Minimally invasive techniques have largely supplanted open cranial vault remodeling as the preferred treatment approach for infants with single-suture craniosynostosis [1–3]. Various strategies have been used to reduce blood loss associated with these procedures, including electrocautery [1], ultrasonic bone-cutting devices [4, 5], preoperative administration of erythropoietin (EPO) and iron therapy [6], tranexamic acid (TXA) [7, 8], and the use of cell salvage systems [9]. Despite their effectiveness, each of these methods has inherent limitations. We present a refined technique for minimizing blood loss during endoscopic strip craniectomy, using a high-speed drill with a diamond burr [10] in combination with a novel attachable dural retractor. The accompanying video illustrates the application of this method in an endoscopic metopic suturectomy. The diamond burr generates heat that cauterizes bone edges, thereby reducing bleeding, while the dural retractor is designed to limit heat dissipation and safeguard the dura from thermal or mechanical injury.

## Methods

### Design of a novel dural retractor

**Fig. 1:**
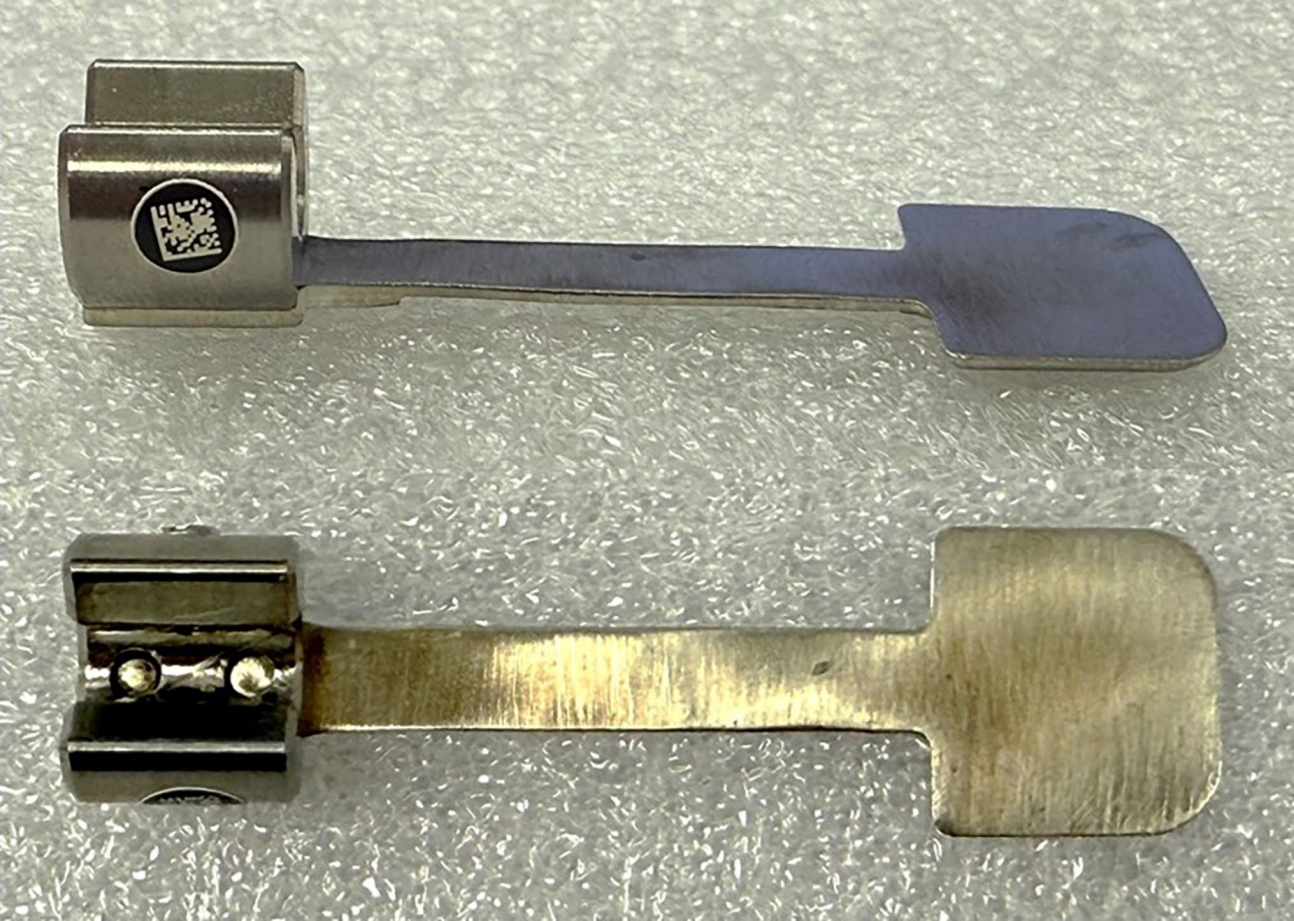
The dural retractor is an attachable device with screws that are flush with its surface

**Fig. 2:**
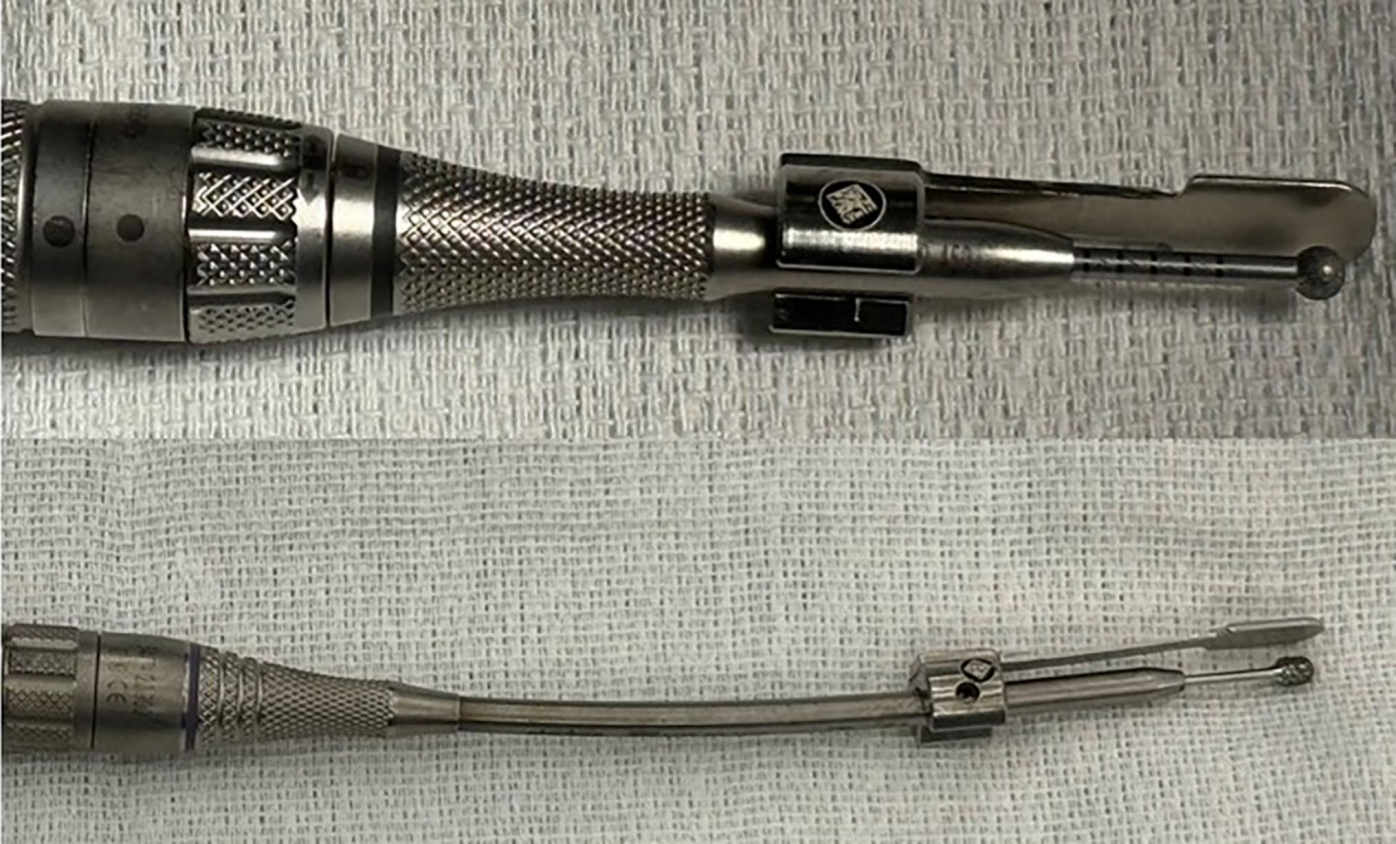
The dural protector attached to both short-straight (top) and long-curved drills (bottom). The short-straight protector is used for the proximal portion of the craniectomy and the long-curved is used for the distal portion

The dural retractor consists of a thin, mildly pliable aluminum strip that attaches securely to a standard surgical drill (Fig. 1). The distal 1 cm of the retractor, aligned with the drill bit, measures 10 mm in width, while the proximal portion beneath the drill shaft has a narrower profile, allowing smooth passage through a 10-mm-wide craniectomy. The attachment screws are recessed and site flush with the device surface to prevent interference during drilling. The retractor is available in two widths (10 and 15 mm) and two lengths, accommodating both short-straight drills and long-curved drill configurations (Fig. 2).

### Clinical details

This is a 3-month-old female, born at 34 weeks gestation, who presented to the craniofacial clinic for evaluation and management of metopic craniosynostosis. On examination, she exhibited prominent trigonocephaly and hypotelorism. The anterior fontanelle was soft and sunken. Her developmental milestones were appropriate for her corrected age, and her neurological examination was normal. After thorough discussion, the parents elected to proceed with an endoscopy-assisted metopic strip craniectomy followed by postoperative helmet therapy.

### Procedure details

**Fig. 3:**
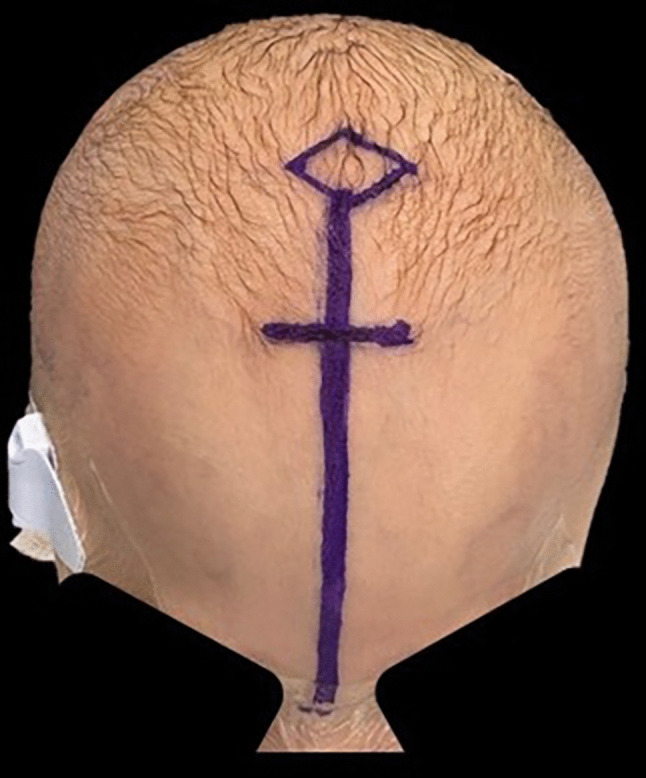
A 2-cm transverse incision is made at the hairline, and the skin is marked to delineate the planned metopic suture craniectomy extending from the anterior fontanelle to nasion

The patient was positioned supine with the head in neutral on a soft horseshoe. An arterial line, foley catheter, and intravenous peripheral lines were placed. TXA was administered per our institutional protocol. Axiem® stealth neuronavigation (Medtronic, Minneapolis, MN) was registered to the preoperative CT scan and accuracy was confirmed using anatomic landmarks to ensure precise midline alignment and accurate nasion identification. A 2-cm transverse incision at the hairline was made over the metopic suture and dissected down to the pericranium (Fig. 3). The galea was elevated off the pericranium along the metopic suture; to the nasion anteriorly and to the anterior fontanelle posteriorly. A burr hole was created using a standard 3.0-mm Carbide Match Head (Stryker, USA) to prevent dural injury. The dura was carefully dissected off the bone. The bit was then replaced with a 4.0-mm round fluted soft touch Diamond bit® (Stryker, Kalamazoo, MI) equipped with the dural retractor device. The metopic suture was drilled in a piecemeal fashion, maintaining a width of 8–10 mm from the nasion anteriorly to the anterior fontanelle posteriorly. The initial portion was drilled using a short-straight attachment (Stryker, USA), while the more distal part of the suture was completed with a long-angled Elite® attachment and a 4.0-mm Coarse Diamond bit® (Stryker, Kalamazoo, MI). To promote bone heating and minimize bleeding, no irrigation was applied during the drilling process. The procedure resulted in minimal blood loss reported as less than 5 cc (pre-op Hgb 10.6 and post-op 10.9), with excellent retraction and visualization achieved using the endoscope (Video 1). The complete extent of the suturectomy was verified by endoscopic visualization, stealth neuronavigation, and palpation of the anterior fontanelle and nasion. No evidence of dural discoloration, shrinkage, or injury was observed intraoperatively. The wound was closed with 4-0 Vicryl sutures in the galea followed by 4-0 Monocryl and steri-strips at the skin. The patient was discharged home POD 1 without complications, required no blood transfusion, and experienced no postoperative complications suggestive of thermal injury.

## Discussion

We report our surgical technique using a diamond drill and attachable dural retractor during endoscopic strip craniectomy for craniosynostosis. The diamond drill is used for its ability to generate localized heat, which in this case appeared to cauterize the drilled bone edges and facilitate hemostasis. In contrast, other commonly used instruments for suturectomy, including high-speed drills with traditional cutting bits, scissors, and rongeurs, often necessitate frequent pauses to control bone-edge bleeding with monopolar electrocautery, bone wax, or a topical hemostatic agent. To preserve the hemostatic effect of localized thermal energy, irrigation was intentionally avoided during drilling, as cooling of the burr may diminish bone-edge cauterization and increase bleeding. The attached blunt dural retractor was designed to serve as a mechanical barrier between the drill and the underlying dura, with the goal of limiting both thermal transmission and inadvertent mechanical injury. In this case, no evidence of dural injury was observed. As with all suturectomy techniques, meticulous dissection of the dura from the inner table of the skull prior to drilling remains essential to minimize the risk of complications.

## Limitations

This report represents a single illustrative case and is therefore limited by its descriptive nature. No quantitative comparison of estimated blood loss, perioperative hemoglobin changes, or transfusion requirements was performed, and conclusions regarding effectiveness cannot be generalized. Although the diamond burr appeared to reduce bleeding from the bone edges intraoperatively, this observation remains subjective and requires validation in larger cohort studies with objective outcome measures. Furthermore, while using a diamond bit to drill a cranial suture may reduce bone-edge bleeding during endoscopic surgery, it does not eliminate the risk of bleeding from the dura or venous sinuses resulting from direct surgical injury.

The deliberate avoidance of irrigation to promote localized bone heating introduces theoretical concerns regarding thermal injury. Although no evidence of dural injury was observed in this case, the protective effect of the attachable dural retractor has not been objectively evaluated through temperature monitoring or histologic assessment. Additional study is necessary to confirm its safety profile.

There is also a technical learning curve associated with mastering controlled and efficient drill use in confined endoscopic corridors. Inexperienced use could potentially negate the intended hemostatic benefit or increase procedural risk. Finally, the dural retractor described in this report is a custom-designed device developed by the senior authors and is not currently commercially available, which limits reproducibility and external validation. Discussions with industry partners regarding potential larger-scale production are ongoing; however, we recognize that the relatively small market size within pediatric craniofacial surgery presents practical challenges to commercialization. Broader clinical adoption would therefore require standardized manufacturing and further prospective evaluation.

## Conclusion

A diamond bit equipped with an attachable dural retractor may reduce bleeding while minimizing dural injury during endoscopy assisted strip craniectomy for craniosynostosis repair. Further evaluation in larger cohort studies is necessary to objectively assess safety, reproducibility, and impact on perioperative blood loss.

## Supplementary Information

Below is the link to the electronic supplementary material.

ESM 1 (MOV 216 MB)

## Data Availability

No datasets were generated or analysed during the current study.
